# Aboveground versus soil‐mediated effects of an invasive grass on fire‐dependent forbs in an oak woodland

**DOI:** 10.1002/ece3.11712

**Published:** 2024-07-17

**Authors:** G. L. Williams, J. Stephen Brewer

**Affiliations:** ^1^ Department of Biology University of Mississippi Oxford Mississippi USA; ^2^ Present address: USDA Forest Service, Pacific Northwest Research Station, Forest Science Lab Corvallis Oregon USA

**Keywords:** aboveground competition, competition–productivity relationship, ecological restoration, invasion ecology, legacy effects, mechanisms of competition, *Microstegium vimineum*, plant–soil (belowground) interactions

## Abstract

Most work on plant competition intensity in general has focused on how aboveground and belowground competition for resources between plants changes with soil resource availability. In contrast, much work on the competitive effects of non‐native invasive species on native species has focused on other mechanisms (e.g., allelopathy and microbial changes) and has largely ignored how these effects interact with mechanisms of resource competition along productivity gradients. We examined aboveground effects of an invasive grass, *Microstegium vimineum*, along with soil differences between invaded and non‐invaded areas on two native perennial herbs at a productive and an unproductive oak woodland site in north Mississippi, USA. We transplanted 32 individuals each of *Helianthus silphioides* and *Potentilla simplex* from uninvaded areas into natural patches dominated by *M. vimineum* at each of the sites. Each transplant was randomly assigned to a pot with either native soil or soil from around *M. vimineum* roots. Aboveground competition was manipulated by securing *M. vimineum* shoots in a non‐shading position around the transplant. We monitored survival of all transplants weekly in the growing seasons of 2020 and 2021. Transplant survival of *H. silphioides* was lowest in *M. vimineum* soil at the more productive site when *M. vimineum* was not pinned back. Transplant survival of *P. simplex* was lower at the more productive site but was mostly unresponsive to pinning and soil treatments. *Synthesis*. Our results suggest that soil‐mediated legacy effects of an invader may reduce some native species' ability to compete for light at productive sites.

## Graphical abstract

Most work on plant competition intensity has focused on how aboveground and belowground competition for resources between plants changes with soil resource availability, while work on the competitive effects of non‐native invasive species on native species has focused on non‐resource‐related mechanisms (e.g., allelopathy) and has largely ignored how these effects interact with other mechanisms of competition along productivity gradients. We examined aboveground and soil‐mediated legacy effects of an invasive grass, Japanese stilt grass, *Microstegium vimineum*, on two native perennial herbs at a productive and an unproductive oak woodland site in north Mississippi, USA. Transplant survival of the Ozark sunflower, *Helianthus silphioides*, was lowest in *M. vimineum* soil at the more productive site when *M. vimineum* was not pinned back, while transplant survival of the common cinquefoil, *Potentilla simplex*, was lower at the more productive site but did not show a statistically significant response to competition treatments, suggesting that soil‐mediated legacy effects of an invader have the potential to reduce some native species' ability to compete for light, especially at productive sites.

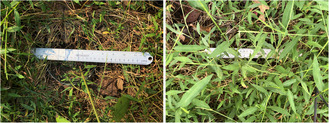

## 1 INTRODUCTION

The intensity and mechanism of plant competition are widely recognized to change with productivity (Grime, 1973; Huston, 2004; Tilman, 1988). Most theoretical and empirical work on competition between plants in general has focused on how aboveground and belowground competition for resources between plants changes with soil resource availability (Brewer, 2003; Cahill, 2002; Twolan‐Strutt & Keddy, 1996; Wilson, 1988; Wilson & Tilman, 1991). There is general agreement that aboveground competition increases with increasing productivity, but how belowground competition changes with productivity is a point of contention (Aerts et al., 1991; Brewer, 2003, 2011a; Twolan‐Strutt & Keddy, 1996; Wilson, 1988; Wilson & Tilman, 1991). Some evidence points to decreased belowground competition with increasing productivity (Emery et al., 2001; Wilson & Tilman, 1991), whereas other evidence has shown that belowground competition increases (Aerts et al., 1991; Wilson, 1988), does not change with increasing productivity (Brewer, 2003; Twolan‐Strutt & Keddy, 1996; Wilson, 1988), or interacts with aboveground competition positively or negatively with increasing soil resource availability (Cahill, 2002; Wilson, 1988).

A general debate over how above‐ and belowground competition changes along productivity gradients (i.e., the Grime–Tilman debate) has largely ignored how competition driven by consumption of resources by plants interacts with other mechanisms of competition (e.g., allelopathic and altered soil microbial communities). Competition between non‐native plants and native plants can occur via the release of toxic compounds into the soil (Callaway & Ridenour, 2004). The negative effects of soil‐mediated changes produced by invaders on native plants often occur belowground (Callaway & Aschehoug, 2000) and may persist even after the invader is removed (soil‐mediated legacy effects). Such effects potentially could occur in productive and unproductive ecosystems. Alternatively, some non‐native species gain a competitive advantage over native species as a result of an evolved growth rate advantage acquired following the loss of chemical defenses (Blossey & Notzold, 1995). An invasive plant with an intrinsic growth rate advantage may crowd or shade slower‐growing and/or shorter native plants (Blossey & Notzold, 1995; Grime, 1977; Huston, 2004). If the growth rate of the invader increases with increasing resource supply, then the intensity of aboveground competition between the invasive plant and the native plant could increase with increasing productivity (Grime, 1977; Huston, 2004). Hence, understanding what gives a non‐native species a competitive advantage is crucial to evaluating the relevance of general competition theory to competition between non‐native and native plants.

When an invasive species can both outgrow and have soil‐mediated legacy effects on native species, then aboveground and belowground effects may interact to influence competitive outcomes in response to site productivity. Soil‐mediated legacy effects that are not directly caused by ongoing soil resource uptake by the invader could still reduce height growth. Thus, multiplicative effects of shading and soil‐mediated factors may result in increased competitive suppression of native species by invasive species at more productive sites. Although previous studies have differentiated aboveground and belowground competitive effects of invasive species on native species (e.g., Gorchov & Trisel, 2003), we are not aware of any previous study that has examined the interaction between growth/size‐mediated aboveground competition and size‐independent soil differences between invasive and native species at sites that differ in productivity.

Japanese stilt grass, *Microstegium vimineum* (Trin.) A. Camus, is a C4 annual introduced to the United States from East Asia that occurs throughout much of the eastern deciduous forests in the United States and Canada (Fairbrothers & Gray, 1972). This non‐native invasive species is one of the most problematic species in the eastern United States from a management perspective (Flory, 2010). *M. vimineum* is highly mycorrhizal, where arbuscular mycorrhizal fungi (AMF) stimulate the invasive plant's growth likely through phosphorus uptake and altered plant architecture (Lee et al., 2014). *M. vimineum* grows at high densities creating monocultures and produces abundant biomass which forms a thick litter layer that decomposes slowly (Ehrenfeld et al., 2001; Flory, 2010). It is significantly more productive in floodplains and lower slopes of uplands than on upper slopes or ridges in uplands (Brewer, 2010, 2011b; Brewer et al., 2015). Native vegetation responds negatively to *M. vimineum* as shown by a reduction in native species richness (Brewer, 2011b; Brewer et al., 2015; Flory & Clay, 2010). While the grass is competitively dominant over native plants, the specific mechanism behind this competitive effect is not known.

Some studies of the effects of *M. vimineum* on native plants have suggested that negative effects are mediated at least partly aboveground (Aronson & Handel, 2011; Brewer, 2011b; Knight et al., 2009; Morrison, 2017; Moyer & Brewer, 2018). On the other hand, because many of the above studies involved complete removal of *M. vimineum* (roots and aboveground parts), none specifically partitioned the competitive effects of *M. vimineum* into their above‐ and belowground effects, and thus one cannot discount the effects of belowground competition. Although some field and greenhouse studies have demonstrated the potential for *M. vimineum* to modify the soil in way that could harm native plants (altered microbial communities, nutrient availability, and allelopathy) (Kourtev et al., 2002; Lee et al., 2012; Pisula & Meiners, 2010; but see Corbett & Morrison, 2012), experimental demonstration of legacy soil effects in the field and their interaction with aboveground competition is lacking.

In this study, we examined aboveground and soil‐mediated negative effects of an invasive grass, *Microstegium vimineum*, on two native perennial herbs at a productive and an unproductive oak woodland site in north Mississippi, USA. We used a factorial arrangement of a pinning‐back treatment and soil origin (*M. vimineum* soil vs. native soil) to determine if and how aboveground competition, soil‐mediated effects, and their interaction varied between two sites that differed in *M. vimineum* productivity. We hypothesized that aboveground competition and soil originating from *M. vimineum*‐dominated patches would have multiplicative, negative effects on the survival of both native species and these effects would be greater at the more productive site (lower slope/small floodplain) than at the less productive site (mid‐to‐upper slope).

## 2 MATERIALS AND METHODS

### 2.1 Study site

This study took place in open oak woodland restoration plots of the upland hardwood forest at the Strawberry Plains Audubon Center, a ~ 1000‐ha wildlife sanctuary located in the loess plains of northcentral Mississippi (USA; 34°49′60″ N, 89°28′32″ W). The restoration plots were managed with the goal of restoring oak woodlands with more open canopies indicative of the historic state of the woodlands prior to extensive logging and fire exclusion, unlike fire‐suppressed oak forests in the surrounding area (Brewer & Menzel, 2009). Two blocks of restoration treatment and control sites (1 ha each), named Wildflower Loop 1 and 2, were established in 2004 and 2017, respectively. The treated site in the older block, Wildflower Loop 1, had undergone both thinning and prescribed burning treatments since 2004, with the latest prescribed burn in the late fall of 2016. The treated site in the more recently established block, Wildflower Loop 2, had undergone canopy thinning since 2017, but not prescribed burning. The treated site at Wildflower Loop 1 likely had lower soil nutrient availability than did the treated site at Wildflower 2, as inferred from lower leaf tissue concentrations in dominant groundcover species at both sites, *Muscadinia rotundifolia* Michx. (M. G. Jewess and J. S. Brewer, unpublished data). Many native plant species increased due to prescribed burning and restoration treatments, but so has *M. vimineum*. Because restoration treatments were started later at Wildflower Loop 2, *M. vimineum* had not yet expanded its population from the lower, wetter portion of the site to upper, drier portions of the site (Brewer et al., 2015). Hence, the large *M. vimineum* patch at Wildflower Loop 2 occurred lower on the slope near an intermittent creek and was more productive than the large patch sampled at Wildflower Loop 1. This was likely due to a shallower fragipan higher on the slope (Providence silt loam) than lower on the slope (Cahaba loam) at these sites with loess parent material on upper slopes and ridges (Tyer et al., 1972). These differences likely corresponded to shallower rooting depths of *M. vimineum* (and other species) higher on the slope than lower on the slope, increasing drought sensitivity higher on the slope (Graveel et al., 2002).

### 2.2 Experimental design

We randomly placed 64, 0.25‐m^2^ plots within a large patch of *M. vimineum* at each of the two treated sites. Each plot was located at least 0.25 m from the closest neighboring plot to ensure that neither shading nor the pinning treatment would interfere with treatments in neighboring plots. In total, 128 plots were established between the two sites. For our target species, we chose two native plant species indicative of fire‐maintained open woodlands that were previously shown to increase in response to the restoration treatments at Wildflower Loop 1: *Helianthus silphioides* Nutt. (Ozark sunflower) and *Potentilla simplex* Michx. (Common cinquefoil) (Brewer et al., 2015). A successful pilot study was conducted over the summer and fall of 2019 to ensure that these species could be transplanted with minimal mortality. Perennial rosettes were documented for all transplanted individuals before transplant removal and cessation of the pilot study in December 2019. Another reason why these two species were chosen is that both were previously shown to respond negatively to *M. vimineum* at Wildflower Loop 1 based on observations over time, in the case of *P. simplex* (Brewer et al., 2015), or through experimental means, in the case of *H. silphioides* (Moyer & Brewer, 2018).

**FIGURE 1:**
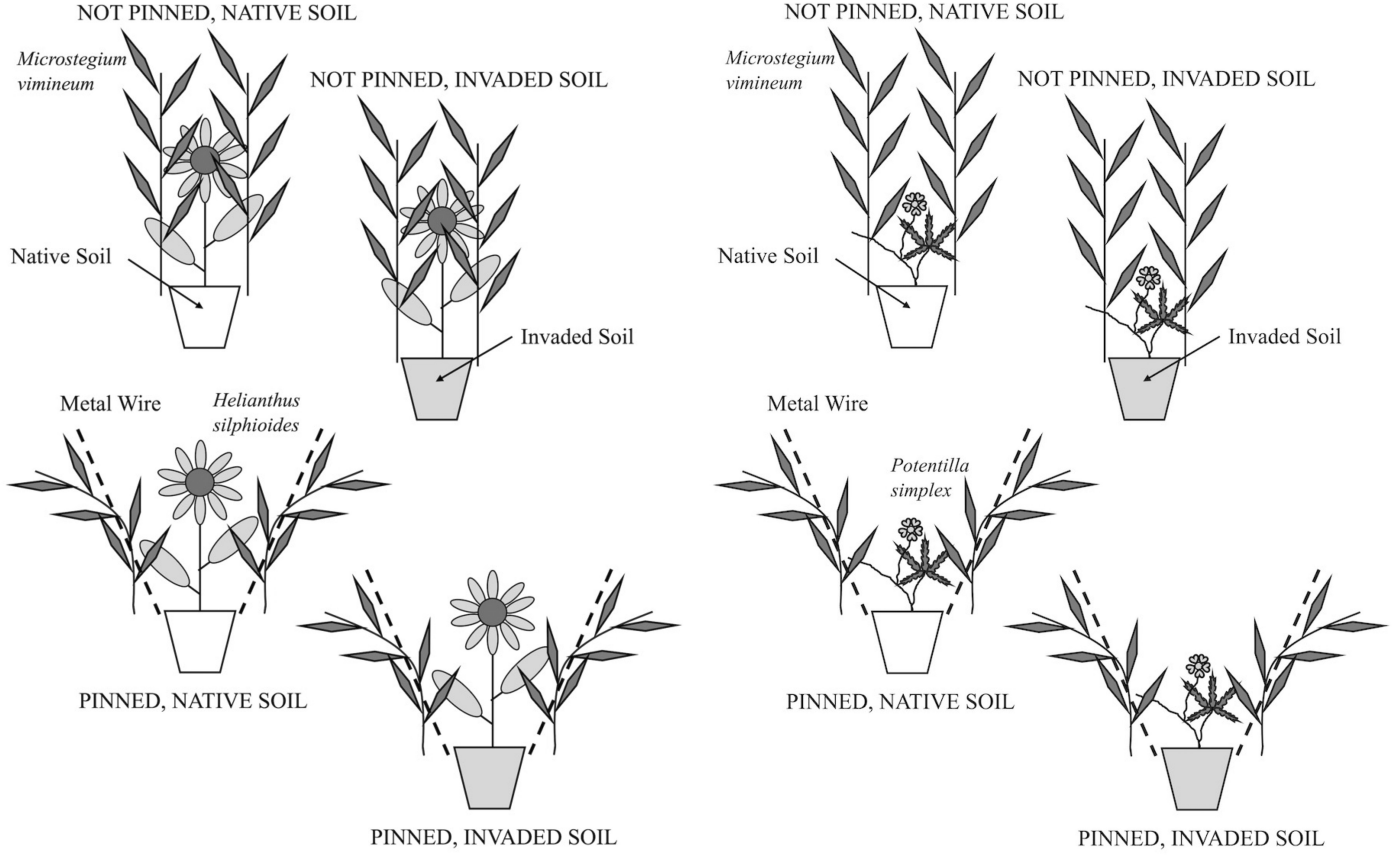
Experimental design showing 8 of 64 subplots in large patches of *Microstegium vimineum* at two sites. One of two native plant species indicative of fire‐maintained open oak woodlands that respond negatively to *M. vimineum* was transplanted in each subplot, for 64 individuals of each native species. Specific treatments for testing an aboveground effect (aboveground *M. vimineum* pinned back or shaded by aboveground *M. vimineum*) and a belowground effect (invaded *M. vimineum* soil or uninvaded, native soil) were randomly assigned to each subplot.

**FIGURE 2:**
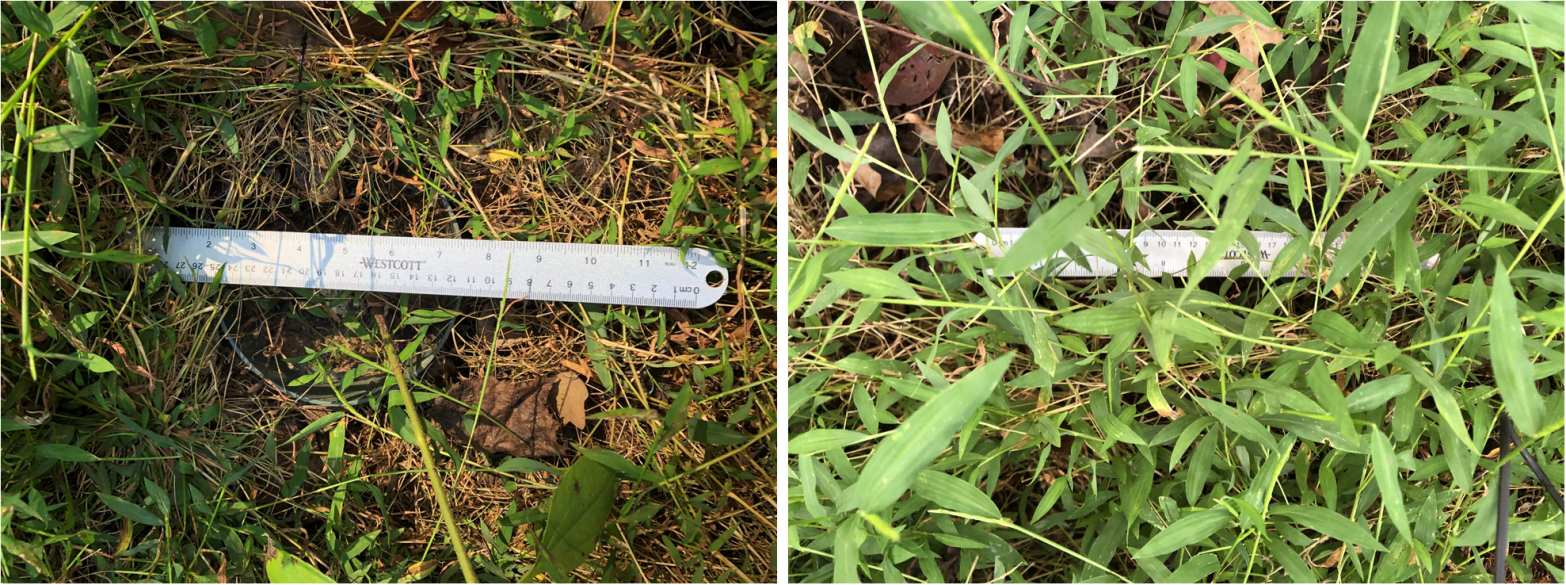
Field photos from September 2020 with a metal ruler over the pot for scale. Photos show the specific treatments for testing an aboveground effect: aboveground *Microstegium vimineum* pinned back (the left panel) or shaded by aboveground *M. vimineum* (the right panel). The wire was effective at pinning back *M. vimineum* shoots to prevent shading, but the wire would occasionally come loose (the left side of the left panel) and need to be resecured to keep the *M. vimineum* pinned. When *M. vimineum* was able to grow naturally, pots assigned to the shading treatment were quickly overgrown by the invader (the right panel).

In November and December 2019, the native plants *H. silphioides* and *P. simplex* were identified and tagged for relocation (transplanting) in early March 2020 (128 plants total). After harvesting, we removed bulk soil from the roots of the rosettes before weighing them to provide an initial size covariate in our statistical analyses. After weighing, we randomly assigned 64 transplants of each of the two species to a 2 × 2 × 2 factorial arrangement of soil origin, aboveground vegetation manipulation, and site treatments (Figure 1). We manipulated soil origin by placing transplants into 15.24‐cm‐diameter, 1.67‐L‐volume pots containing either uninvaded, native soil from Wildflower Loop 1 where the transplants were harvested without *M. vimineum* presence (native soil), or invaded *M. vimineum* soil from the hole dug into each plot in the *M. vimineum* patches for the transplant pot (invaded soil). Ten‐cm‐deep holes were dug into each plot for planting the potted transplants, but the excavated soil was only used in the invasive soil treatments. Excess soil was discarded downslope from each *M. vimineum* patch. Potted transplants were then placed with the rims of the pots level to the topsoil in the holes within the *M. vimineum* patches. After we placed the pots in the ground within the *M. vimineum* patches at each site, we administered the aboveground competition treatment. Within one‐half of the plots, to reduce aboveground competition, we secured *M. vimineum* stalks in the immediate vicinity of the pot in a non‐shading position away from the transplant by metal wires (the pinned back treatment) (Figure 2). We reapplied this treatment as necessary to hold the *M. vimineum* stalks in place throughout the growing season. We did not manipulate *M. vimineum* stalks in the other half of the plots (not pinned back) to allow the *M. vimineum* to grow vertically and shade the transplants (Figure 2). It rained on both transplanting days in 2020, rendering the need for water transplants unnecessary.

### 2.3 Data collection

To quantify the response variable of transplant survival, we monitored all transplanted individuals weekly in the spring and biweekly in the 2020 growing season and from emergence to senescence in 2021 growing season. We quantified cumulative transplant survival over both the 2020 and 2021 growing seasons by counting the number of biweekly censuses in which the transplant was alive, starting with the initial census. Hence, counts ranged from 1 to 12 with a count of 12 corresponding to the individual surviving the entire experiment. We also monitored herbivory throughout the growing season. Although herbivory was present on some of the larger *H. silphioides* individuals toward the end of the field season, there was no evidence of herbivory stunting the growth or flowering of the plant or killing *H. silphioides* transplants, and thus herbivory results are not presented here.

Because *M. vimineum* responds positively to tree canopy gaps (Brewer, 2010), we measured overhead canopy gaps in June 2020 using a canopy densiometer (Forest Densiometers, Rapid City, SD, USA) and the leaf area index (LAI) and diffuse non‐interceptance (DIFN) 1 m above each plot using an LAI‐2000 Plant Canopy Analyzer (LI‐COR biosciences, Lincoln, NE, USA) in June 2020 for possible use (along with initial size) as covariates in the analysis of treatment effects. We did not record soil moisture for each transplant because of concerns about the soil moisture probe damaging the transplants or their roots within each pot and confounding the results of this study. We also recorded the elevation of each plot using a Suunto PM‐5 clinometer (Suunto, Vantaa, Finland) in June 2021. We measured plant reproduction by recording the number of flowering heads on both species, in the spring of 2021 for *P. simplex* and the fall of 2020 and 2021 for *H. silphioides*. End‐of‐season *M. vimineum* productivity for each site was recorded in February 2023 by measuring the overwinter (post‐senescence) heights of *M. vimineum*. Previous work with this species at multiple sites and slope positions indicates plant height is a good indicator of *M. vimineum* productivity (Brewer, 2010; Brewer et al., 2015). The data, metadata, and R scrip for all analyses are available from the Dryad Digital Repository: 10.5061/dryad.zkh1893hb (Williams & Brewer, 2024). (metadata and data files).

### 2.4 Data analysis

We tested the effects of soil origin, pinning, and site on the cumulative survival of transplants with generalized linear models (function *glm*) using the stats package in R 4.1.0 (R Core Team, 2020). Because we had reason to believe that the two sites differed in productivity and were interested in these differences and how they interacted with the treatments, we treated site as a fixed effect rather than a random effect. We recognize, however, that site productivity was not replicated in this study and acknowledge the need for caution when interpreting its effect as a productivity effect. We used the Poisson response distribution (family = “poisson”) when fitting census counts to pinning, soil origin, and site and their interactions. Because both the initial fresh mass of the transplant (initial mass) and canopy openness in June 2020 accounted for non‐neglible amounts of variation in cumulative transplant survival, we included them in the glm as covariates. There were too few observations of flowering individuals to test responses to treatments; therefore, we only present mean counts and standard errors. We analyzed treatment effects on cumulative survival using the *Anova* function (package car) and type 2 sums of squares with the test statistic of “Wald” chi‐square, wherein the main effects of the treatments, site, and the covariates were tested first, then the two‐way interactions among pinning, soil origin, and site, and then the three‐way interaction among pinning, soil origin, and site. We calculated expected marginal means using the *emmeans* function (package *emmeans* (Lenth, 2023)), which reported the mean counts on a log scale. Confidence intervals and standard error bars were derived from the mean squared error for the test of a given effect or interaction, except where noted, in which case we calculated standard error separately for each treatment combination. We tested for site differences in *M. vimineum* productivity (overwinter height) and a possible correlation with these differences (elevation of plots) using linear models. Residuals were normally distributed, and thus we fit untransformed heights and elevation to the predictor, site.

## 3 RESULTS

### 3.1 *Helianthus silphioides* survival and flowering responses

**FIGURE 3:**
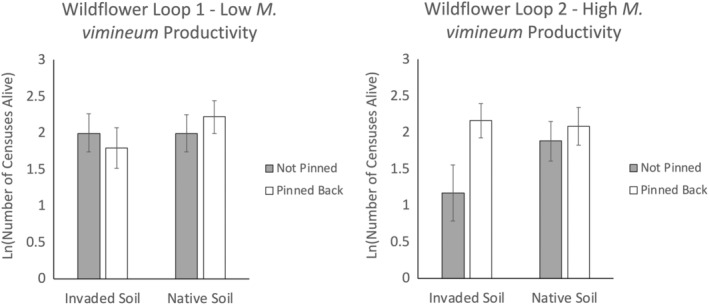
The difference in *Helianthus silphioides* survival by the Soil Origin × Pinning × Site interaction during the 2021 growing season. Values are the mean natural log of cumulative transplant survival maximum possible = ln(12 censuses) = 2.48. Error bars correspond to 95% asymptotic confidence intervals.

**FIGURE 4:**
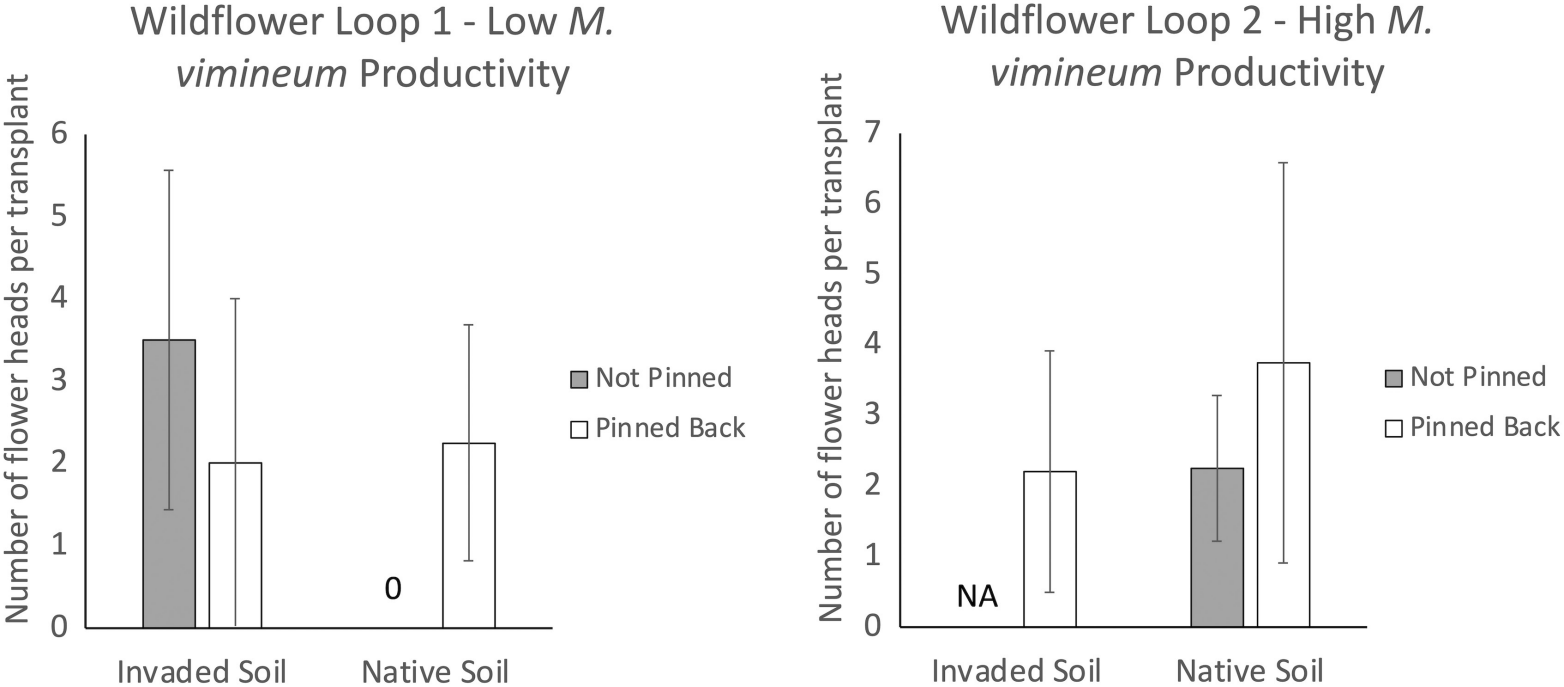
Flower head production by *Helianthus silphioides* with respect to differences in site, pinning, and soil origin during the 2021 flowering season (autumn). Values are the number of heads produced per reproductive transplant. Error bars are ±1 standard error for each combination of site, pinning, and soil origin. The 0 value indicates that no flower heads were produced while the NA value indicates that there were no living transplants to produce flower heads.

Cumulative transplant survival of *H. silphioides* over the 2020 and 2021 growing seasons was lowest in invaded soil at the more productive site when *M. vimineum* was not pinned back, resulting in a significant three‐way interaction among pinning, soil origin, and site (*X* ^2^_(64)_ = 9.352, *p* = .002; Figure 3). The effect size of pinning back *M. vimineum* in invaded soil at the more productive Wildflower 2 site was 1.111 SD compared to only 0.261 SD in the native soil at the same site. In contrast, at the less productive Wildflower Loop 1 site, the effect size of pinning back *M. vimineum* was −0.132 and 0.169 in the invaded soil and native soil, respectively. Interpretation of the main effects and other interactions is therefore not straightforward, but the results of these statistical tests are presented in Table 1. Transplant survival was positively associated with the covariate, initial fresh mass of the transplant (*X* ^2^_(64)_ = 8.021, *p* = .005). The number of flower heads produced per transplant in 2021 was highly variable and exhibited no obvious trends in response to the treatments (Figure 4).

**TABLE 1:** Analysis of deviance table showing how cumulative transplant survival of *Helianthus silphioides* over two growing seasons responded to site, soil, and pinning treatments and their interactions.

Analysis of deviance—type 2 sums of squares			
Response: Number of censuses alive in 2020 and 2021			
	Df	Wald Chi‐square	*p*
Soil Origin—So	1	4.599	.032
Pinning—P	1	6.850	.009
Site—Si	1	1.573	.210
So × P	1	0.308	.579
So × Si	1	0.000	.985
P × Si	1	5.940	.015
So × P × Si	1	9.352	.002
Covariates			
Canopy openness June 2020	1	0.725	.395
Initial transplant mass	1	8.021	.005

### 3.2 *Potentilla simplex* survival responses

**FIGURE 5:**
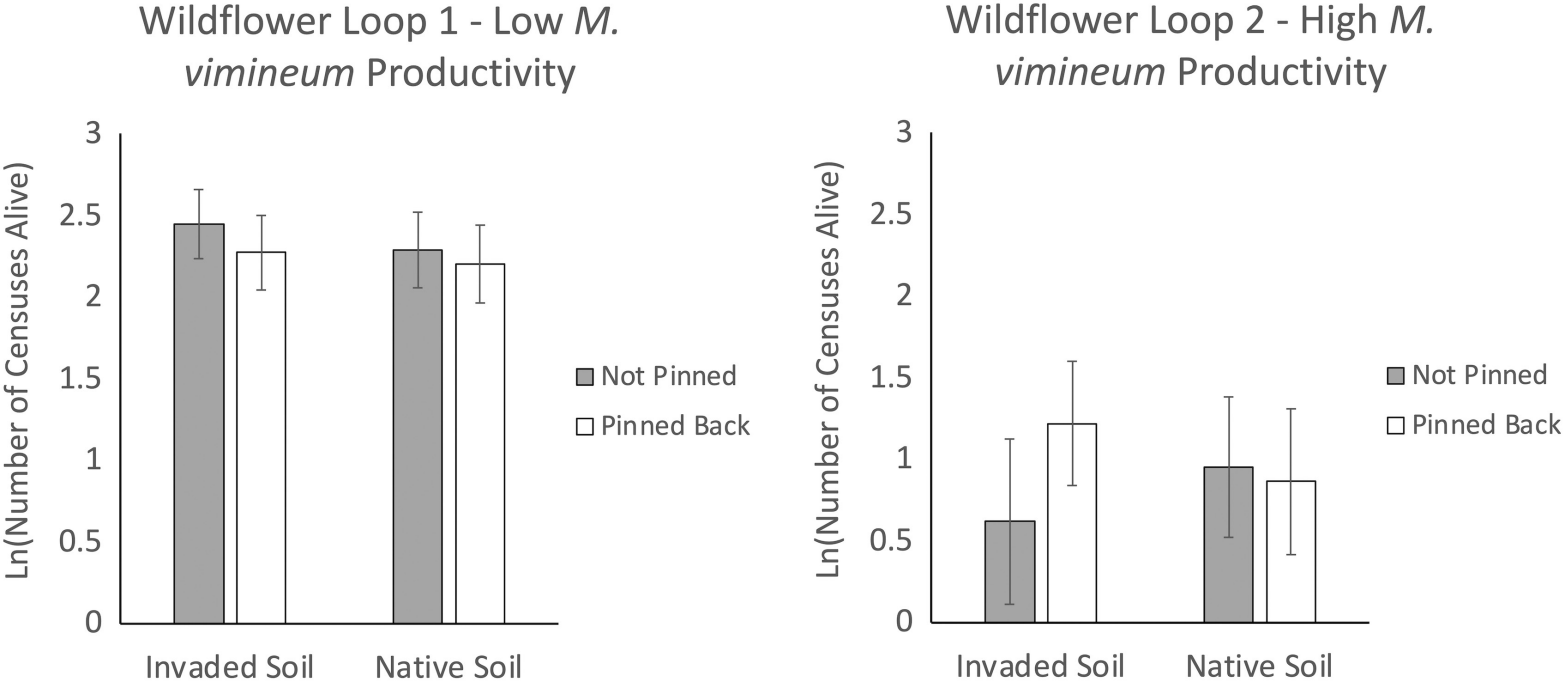
The difference in *Potentilla simplex* survival by the Soil Origin × Pinning × Site interaction during the 2021 growing season. Values are the mean natural log of cumulative transplant survival maximum possible = ln(12 censuses) = 2.48. Error bars correspond to 95% asymptotic confidence intervals.

In contrast to *H. silphioides*, there was no clear evidence of aboveground competitive or soil legacy effects of *M. vimineum* on survival of *P. simplex* in this study, as indicated by the lack of significant main effects of pinning, soil origin, or any interactions (Table 2). Cumulative transplant survival of *P. simplex* over the 2020 and 2021 growing seasons was much higher at the site with low *M. vimineum* productivity than at the site with higher *M. vimineum* productivity (*X* ^2^_(64)_ = 113.343, *p* < .001; Figure 5). Although the three‐way interaction among site, pinning, and soil origin was not statistically significant for *P. simplex* (*X* ^2^_(64)_ = 2.356, *p* = .125), the cumulative survival responses depicted in Figure 5 suggest a weak positive effect of pinning back *M. vimineum* at Wildflower Loop 2 when *P. simplex* was grown in invaded soil (effect size = 0.469), but not in native soil (effect size = −0.069). There was no positive effect of pinning in either invaded soil (effect size = −0.276) or native soil (effect size = −0.131) at Wildflower Loop 1 (Figure 5).

**TABLE 2:** Analysis of deviance table showing how cumulative transplant survival of *Potentilla simplex* over two growing seasons responded to site, soil, and pinning treatments and their interactions.

Analysis of deviance—type 2 sums of squares			
Response: Number of censuses alive			
	Df	Wald Chi‐square	*p*
Soil Origin—So	1	1.012	.315
Pinning—P	1	0.277	.599
Site—Si	1	113.343	<.001
So × P	1	0.103	.748
So × Si	1	0.078	.781
P × Si	1	2.336	.126
So × P × Si	1	2.356	.125
Covariates			
Canopy openness June 2020	1	0.024	.876
Initial transplant mass	1	1.661	.198

### 3.3 Site differences in the productivity of *M. Vimineum* and plot elevation

*M. vimineum* productivity, measured as end‐of‐season plant height for this annual plant, was much greater at Wildflower Loop 2 than at Wildflower Loop 1 (118.920 ± 6.227 cm vs. 65.58 ± 4.186 cm for Wildflower Loop 2 and Wildflower Loop 1; *F*_(1,18)_ = 50.353, *p* < .001). The two sites also differed with respect to average elevation above sea level of the plots, which was lower at Wildflower Loop 2 than at Wildflower Loop 1 (147 ± 0.0782 m vs. 154.4 ± 0.111 m for Wildflower Loop 2 and Wildflower Loop 1, respectively; *F*_(1,126)_ = 4275, *p* < .001). The lower elevation of the plots at Wildflower Loop 2 corresponded to their being located in a small floodplain associated with an intermittently running creek at the base of a slope.

## 4 DISCUSSION

The results of our study provide partial support for the hypothesis that aboveground competition and soil legacy effects from an invasive grass can interact to influence the survival of native herbaceous plants. In particular, we found that a regionally endemic sunflower, *H. silphioides*, was suppressed by *M. vimineum* when *H. silphioides* was grown in soil taken from areas dominated by *M. vimineum* and was subjected to aboveground competition from *M. vimineum* at a site at which *M. vimineum* was productive and tall. We suggest that soil legacy effects of *M. vimineum* made *H. silphioides* more vulnerable to shading by taller plants of *M. vimineum*. We suspect that the ability of *M. vimineum* to grow taller at Wildflower Loop 2 than at Wildflower Loop 1 was due to the fact the large patch of *M. vimineum* at Wildflower Loop 2 occurred farther downslope and was located in the floodplain of a small creek. Hence, greater soil moisture associated with more frequent flooding and/or greater, unimpeded root growth due to the lack of a shallow fragipan lower on the slope could have contributed to the greater productivity of *M. vimineum* at Wildflower Loop 2 (Graveel et al., 2002; Tyer et al., 1972). Previous work has shown that *M. vimineum* colonizations, spread rate, and invasion success were found to be higher at more mesic sites than at more xeric sites (Brewer, 2010; Huebner, 2010), suggesting that site productivity differences are a major factor affecting the invasion success, and likewise the competitive ability, of *M. vimineum*. The interactive effects of aboveground competition and soil legacy on the other native species examined, *Potentilla simplex*, are not clear. Although *P. simplex* showed much lower survival at Wildflower Loop 2 than at Wildflower Loop 1, we do not have convincing evidence that the lower survival of *P. simplex* at the more productive site for *M. vimineum* was due to more intense competition between *M. vimineum* and *P. simplex*. Rather, *P. simplex* might not have been able to tolerate the more frequent flooding that occurred at Wildflower Loop 2 than at Wildflower Loop 1. That said, although the three‐way interaction of site, pinning, and soil origin was not statistically significant for *P. simplex* (*X* ^2^_(64)_ = 2.356, *p* = .125), the survival of *P. simplex* was lowest at Wildflower Loop 2 for plants grown in soil invaded by *M. vimineum*, when *M. vimineum* was not pinned back (Figure 5).

Although we suspect that negative effects of soil invaded by *M. vimineum* were the result of legacy effects of occupation by *M. vimineum*, we cannot rule out the possibility that invaded soils were less suitable for *H. silphioides* survival but more suitable for *M. vimineum* invasion prior to its invasion. We think this alternative explanation is less likely for the following reasons. First, previous work has shown that *H. silphioides* and *M. vimineum* have both increased in response to restoration treatments (canopy reduction and fire) (Brewer et al., 2015). Second, there is no evidence that patch expansion of *M. vimineum* is in any way restricted by biotic resistance from the resident plant community (Brewer et al., 2015; Williams & Brewer, 2024). Likewise, there is no evidence of lasting competitive suppression of *M. vimineum* by *H. silphioides* (Moyer & Brewer, 2018). Rather, expanding patches of *M. vimineum* simply displaced native species, including *H. silphioides* (Brewer et al., 2015; Moyer & Brewer, 2018; Williams & Brewer, 2024). Altogether, these observations strongly suggest that *M. vimineum* alters the soil in way to make it less suitable for survival of *H. silphioides*, when it is also heavily shaded by *M. vimineum* at productive sites.

The results of this field experiment highlight the importance of competition studies that examine more than one mechanism of competition when multiple mechanisms have been proposed. For plants in general, increased competition intensity with increasing productivity is generally attributed to increased competition for light or increased competition for light and soil resources (Aerts et al., 1991; Twolan‐Strutt & Keddy, 1996; Wilson & Tilman, 1991). In contrast, the negative effects of soil‐mediated changes produced by invaders on native plants often occur belowground (Callaway & Aschehoug, 2000) and are not necessarily related to resource supply or productivity. Our results partially agree with those of previous studies showing that aboveground competition is highest when productivity is high (Aerts et al., 1991; Twolan‐Strutt & Keddy, 1996; Wilson, 1988; Wilson & Tilman, 1991), but are also consistent with the hypothesis that the effects of a competitor aboveground are not independent of its effects belowground or on the soil (Cahill, 2002). Hence, understanding the impacts of invasive plants on native plants may require examining how both resource availability and non‐resource‐related legacy effects in the soil combine to influence competitive interactions along productivity gradients.

Most previous discussions of the interaction between aboveground and belowground competition have emphasized the potential for competition for light to interact with competition for soil resources (i.e., nutrients and/or water) (Cahill, 2002; Wilson, 1988). We suggest that such an interaction in resource competition was unlikely in our experiment for three reasons. First, belowground competition for soil resources between *M. vimineum* and the native transplants was not allowed to occur during our study. Transplants were grown in pots, and the roots of neighboring *M. vimineum* plants were not permitted access to the soil around the transplants during the study. Thus, it was not possible for increased *M. vimineum* productivity at Wildflower Loop 2 to result in increased root competition for soil resources at this site. Second, although it is possible that the roots of *M. vimineum* depleted soil nutrients prior to transplanting, previous work with this species suggests that it increases rather than decreases soil nitrogen availability (Ehrenfeld et al., 2001). If *M. vimineum* increased soil nutrient availability in soils at our sites, then we cannot think of a reason why the survival of *H. silphioides* transplants would be reduced in such soils in the absence of competition from *M. vimineum*. Third, although it is possible that the roots of *M. vimineum* depleted soil moisture prior to transplanting, the greatest negative effect of *M. vimineum* soil on *H. silphioides* survival was at Wildflower Loop 2, the more frequently flooded site. Site differences in hydrology continued during the study. Inadequate soil moisture therefore seems an unlikely explanation for the low survival of shaded *H. silphioides* transplants in *M. vimineum* soils at Wildflower Loop 2. Although we suspect that the soil‐mediated legacy effects (e.g., allelopathy and losses of microbial mutualists) combined with competition for light increases the negative impact of *M. vimineum* on *H. silphioides* at the more productive site, the exact cause of such soil‐mediated negative effects remains unclear. Greenhouse studies have reported that *M. vimineum* likely alters the soil environment and exhibits the potential for allelopathic and/or other belowground effects on native vegetation in the field. A study comparing the belowground effects of *Berberis thunbergii* DC. and *M. vimineum* discovered that the two invasive species, from a location of uniform land‐use history and canopy cover but sampled from adjacent soils, contained pronounced differences in their soil microbial communities (Kourtev et al., 2002). A greenhouse experiment on the allelopathic potential of bioassays of 10 invasive plants on radishes (*Raphanus sativus* L.) found *M. vimineum* to produce a large inhibitory effect on the target species (Pisula & Meiners, 2010). Similarly, *Lactuca sativa* L. seed germination was inhibited by aqueous solutions of the aboveground tissues of *M. vimineum* in a greenhouse (Speigel III & Morrison, 2015). In contrast, *M. vimineum*'s allelopathic potential using whole‐plant aqueous extracts on a native plant found that the allelopathic potential of the invader was not greater than that of co‐occurring native species (Corbett & Morrison, 2012). As such, there is not a current consensus in the literature on the allelopathy of *M. vimineum*.

The observed competitive response of *H. silphioides* to *M. vimineum* provides one possible mechanism to explain the previous observations of species richness losses associated with patch expansion of *M. vimineum* in this system (Williams & Brewer, 2024; Brewer et al., 2015). Both of these previous community‐level studies found increased rates of loss of native species diversity following more rapid rates of patch expansion of *M. vimineum* on more productive lower slopes of oak woodlands. Hence, these observations are consistent with the hypothesis that competitive displacement of species by invaders is greater in areas in which the invader is more productive (Huston, 2004). We suggest, however, that multiplicative effects of soil legacies and shading produced by invasive species could be an even more potent force for eliminating native species than competition for light alone, one that potentially increases as the supply of resources to the invader increases.

## AUTHOR CONTRIBUTIONS

**G. L. Williams:** Conceptualization (equal); data curation (equal); formal analysis (equal); investigation (equal); methodology (equal); resources (equal); visualization (equal); writing – original draft (lead); writing – review and editing (equal). **J. Stephen Brewer:** Conceptualization (equal); data curation (equal); formal analysis (equal); investigation (equal); methodology (equal); resources (equal); supervision (lead); visualization (equal); writing – original draft (supporting); writing – review and editing (equal).

## CONFLICT OF INTEREST STATEMENT

The authors declare that they have no conflict of interest.

## Data Availability

The data and appendix that support the findings of this study are available from the Dryad Digital Repository: 10.5061/dryad.zkh1893hb (Williams & Brewer, 2024).
